# CircZfp644-205 inhibits osteoblast differentiation and induces apoptosis of pre-osteoblasts via sponging miR-455-3p and promoting SMAD2 expression

**DOI:** 10.1186/s40001-024-01903-7

**Published:** 2024-06-08

**Authors:** Peng Zhang, Jie Liu, Zijia Chai, Jinjin Fu, Shuwen Li, Zhe Yang

**Affiliations:** 1 Department of Orthopaedics, Shanxi Provincial People’s Hospital, No.29, Shuangta Temple Street, Taiyuan, 030012 Shanxi China; 2 Department of Internal Neurology, Inner Mongolia Medical University Affiliated Hospital, Hohhot, Inner Mongolia China; 3 Shenzhen University General Hospital, Shenzhen, Guangdong China; 4 Heze Municipal Hospital, Heze, Shandong China; 5 Department of Minimal Invasive Spine Surgery, The Second Affiliated Hospital of Inner Mongolia Medical College, Hohhot, Inner Mongolia China

**Keywords:** Osteoblast differentiation, circRNA, miRNA, ceRNA, Apoptosis, SMAD2

## Abstract

**Background:**

Circular RNAs (circRNAs) are involved in the progression of osteoporosis; however, their impact on osteogenic differentiation has yet to be fully elucidated. In this study, we identified a novel circRNA known as circZfp644-205 and investigated its effect on osteogenic differentiation and apoptosis in osteoporosis.

**Methods:**

CircZfp644-205, miR-445-3p, and SMAD2 levels were measured using quantitative real-time polymerase chain reaction (qRT-PCR). MC3T3-E1 cells were subjected to microgravity (MG) to establish a cell model. Osteogenic differentiation was assessed using qRT-PCR, Alizarin Red S staining, alkaline phosphatase staining, and western blot. The apoptosis was evaluated using flow cytometry. The relationship between miR-445-3p and circZfp644-205 or SMAD2 was determined using bioinformatics, RNA pull-down, and luciferase reporter assay. Moreover, a hindlimb unloading mouse model was generated to investigate the role of circZfp644-205 in vivo using Micro-CT.

**Results:**

CircZfp644-205 expression was up-regulated significantly in HG-treated MC3T3-E1 cells. Further in vitro studies confirmed that circZfp644-205 knockdown inhibited the osteogenic differentiation and induced apoptosis of pre-osteoblasts. CircZfp644-205 acted as a sponge for miR-455-3p, which reversed the effects of circZfp644-205 on pre-osteoblasts. Moreover, miR-455-3p directly targeted SMAD2, thus inhibiting the expression of SMAD2 to regulate cellular behaviors. Moreover, circZfp644-205 alleviated the progression of osteoporosis in mice.

**Conclusions:**

This study provides a novel circRNA that may serve as a potential therapeutic target for osteoporosis and expands our understanding of the molecular mechanism underlying the progression of osteoporosis.

## Introduction

Osteoporosis is primarily characterized by decreased bone mass and disruption of bone microstructure [1]. With the development of the aging population, the prevalence of osteoporosis and its associated complications has risen to 13.2% due to the aging population, seriously affecting the health and well-being of elderly individuals. The dynamic balance of osteoblasts and osteoclasts is crucial for maintaining bone tissue homeostasis. Several factors, including nutrition, hormone levels, age, and lifestyle, can influence this balance. Disruption of this balance can lead to a range of bone disorders, including osteoporosis. Osteoblasts play a critical role in bone formation and repair [2]. Thus, understanding the molecular mechanism of osteoblast-mediated osteogenesis may contribute to identifying novel targets for the treatment of osteoporosis.

Noncoding RNAs regulate critical signaling molecules associated with the progression of osteoporosis progression and other bone diseases [3]. Circular RNAs (circRNAs) are a new subgroup of noncoding RNAs characterized by a covalently closed loop lacking 5ʹ–3ʹ polyadenylated tails, typically forming through back-splicing events. Studies have demonstrated that circRNAs are highly conserved, abundant, and possess high stability across different species [4]. Moreover, increasing evidence suggests that circRNAs participate in various physiological and pathophysiological processes, including modulating alternative splicing as microRNA (miRNA) sponges and regulating protein–RNA interactions to affect cell phenotype. Numerous studies have concentrated on circRNAs as miRNA sponges, wherein miRNAs subsequently bind to the 3ʹ untranslated region (3ʹUTR) of target genes to regulate cellular biological functions. Previous studies have revealed the critical role of the circRNA/miRNA/target gene axes in the occurrence and/or progression of osteoporosis. For example, circRNA_0016624 can prevent the development of osteoporosis via sponging miR-98 and elevating the expression level of BMP2 [5]. Besides, circRNA_0006393 can promote glucocorticoid-induced osteoporosis through regulating the miR-145-5p/FOXO1 axis [6]. Exosomes derived from circRNA Rtn4-modified BMSCs inhibit TNF-α-induced apoptosis of MC3T3-E1 cells by interacting with miR-146a [7]. In addition, circRNAs serve as potential prognostic and diagnostic biomarkers for osteoporosis, such as circ_0002060 and circ_0076690 [8, 9]. However, the regulatory network of circRNAs in osteoporosis is intricate, and the functions and molecular mechanisms have not yet fully elucidated.

Bone interacts with muscle through physical forces, including gravity. Cellular biological behaviors are altered after sensing mechanical forces, leading to bone formation [10]. The lack of gravitational forces leads to injury of bone microarchitecture, which is clinically defined as osteoporosis [11]. Due to the bone loss observed in skeletons exposed to microgravity (MG) exposure [12], in the study, we stimulated MC3T3-E1 cells with MG to establish a cell model. A novel circRNA, circZfp644-205, was identified and found to be up-regulated in MG-treated cells. Therefore, this study aimed to investigate the effects of circZfp644-205 on osteogenic differentiation and apoptosis of pre-osteoblasts, as well as the underlying mechanism. Moreover, the effect of circZfp644-205 on osteoporosis progression was explored in hindlimb unloading mice, an animal model of osteoporosis that inhibits bone formation [13]. This study will provide a novel target for the treatment of osteoporosis.

## Methods

### Cell culture

The mouse cranial osteoblast MC3T3-E1 cells were obtained from the Shanghai Institute of Biochemistry and Cell Biology (Shanghai, China) and cultured in Dulbecco’s modified Eagle medium (Hyclone, Logan, USA) containing 10% fetal bovine serum (FBS, Gibco, Carlsbad, USA), 100 mg/mL streptomycin, and 100 U/mL penicillin (Solarbio, Beijing, China). For osteogenic differentiation, MC3T3-E1 cells were cultured in a specific culture medium containing 100  nM dexamethasone, 50 μM ascorbic acid, and 10 mM β-glycerophosphate (Sigma, USA) for 14 days.

### Simulated microgravity

To establish the cell model to mimic osteoporosis, the cells were subjected to microgravity stimulation using a 2D clinostat (developed by the China Astronaut Research and Training Center). Briefly, MC3T3-E1 cells (1 × 10^5^) were seeded on cell climbing pieces. After culturing for 24 h, the climbing pieces were placed in a box 12.5 mm away from the rotational axis. Then, after completely removing the bubbles, the container cover was tightly screwed down. Subsequently, the boxes were fixed in the clinostat and rotated around a horizontal axis at 30 rounds per min (rpm) for 10 min in the cell incubator at 37 ℃ with 5% CO_2_. The vertical rotation group was used as the control.

### Cell transfection

MC3T3-E1 cells were seeded in six-well plates and grown until approximately 80% cell confluence. CircZfp644-205 overexpression vector (circZfp644-205), empty vector, small interfering RNA (siRNA) targeting circZfp644-205 (si-circZfp644-205), siRNA targeting SMAD2 (si-SMAD2), siRNA negative control (si-NC), miR-455-3p mimic, mimic NC, miR-455-3p inhibitor, and inhibitor NC were obtained from GenePharma (Shanghai, China). They were transfected into MC3T3-E1 cells using Lipofectamine 2000 (Invitrogen, USA). After 48 h, the cells were harvested.

### Alizarin Red S (ARS) and alkaline phosphatase (ALP) staining

The osteogenic differentiation capability was evaluated using ARS and ALP staining assays. For ARS staining, after treatment, MC3T3-E1 cells were fixed in 4% paraformaldehyde (Aladdin, Shanghai, China) for 10 min, washed with distilled water, and then stained with 1% ARS solution in accordance with the manufacturer's protocols (Leagene, Beijing, China). Finally, the stained cells were rinsed with distilled water, and the matrix calcification with red deposition was observed using a microscope (Nikon, Tokyo, Japan).

For ALP staining, MC3T3-E1 cells were fixed in 70% ethanol for 1 h and washed three times with distilled water. ALP staining was performed using an ALP staining kit (Sidansai, Shanghai, China) according to the manufacturer's protocols. Then, the stained cells were photographed by a microscope (Zeiss, Oberkochen, Germany). The absorbance of stained cells was observed at 562 nm on a microplate reader (BioTek, Winooski, USA) to quantify ALP staining intensity.

### Bioinformatics analysis

The DIANA-TarBase database (version 8.0; http://diana.imis.athena-innovation.gr/DianaTools) [14] was used to predict the target genes of miR-455-3p.

### Flow cytometry

Cell apoptosis was analyzed using flow cytometry. The cells, 48 h post-transfection, were washed thrice with PBS. Subsequently, the cells were stained with 2.5 g/mL of Annexin V-FITC and propidium iodide (PI). Finally, apoptosis was evaluated using a flow cytometer (FACSVerse, BD, USA), and the data were analyzed with the FlowJo software (v.7.0, FlowJo LLC).

### Quantitative real-time polymerase chain reaction (qRT-PCR)

Total RNA was extracted with Trizol reagent (Invitrogen, USA). RNA samples were transcribed into cDNA using the Superscript II kit (Beyotime, Shanghai, China). Specific primers were designed and synthesized by General BIO (Anhui, China). SYBR Green mix (Takara, Dalian, China) and ABI 7500 thermal circulator (Applied Biosystems, USA) were used to detect the relative level of target genes. The thermocycling conditions were as follows: initial denaturation at 95 ℃ for 10 min, followed by 40 cycles of 95 ℃ for 15 s and 60 ℃ for 1 min. U6 and GAPDH served as the loading controls for miRNA and mRNA, respectively. The expression levels were determined using the 2^−ΔΔCt^ methods. The primers used are as follows (5ʹ– > 3ʹ):

circZfp644-205: Forward, TCAAAAGAACTGGCAACGGG; Reverse, ACGCTTGTGGTCTTTCTCGT.

Linear Zfp644: Forward, GGCCATGGAAGAAGAAGCCT; Reverse, ATCGTAACTCAGGGCAGCAC.

Runx2: Forward, TCAACGATCTGAGATTTGTGGG; Reverse, GGGGAGGATTTGTGAAGACGG.

Bglap: Forward, GGCGCTACCTGTATCAATGG; Reverse, GTGGTCAGCCAACTCGTCA.

COL1A1: Forward, GAGGGCCAAGACGAAGACATC; Reverse, CAGATCACGTCATCGCACAAC.

ALP: Forward, ACCACCACGAGAGTGAACCA; Reverse, CGTTGTCTGAGTACCAGTCCC.

MiR-455-3p: Forward, GCAGTCCATGGGCATATACAC; Reverse, GGGTGGGCCAGGCTGTGGGGCG.

SMAD2: Forward, CCGACACACCGAGATCCTAAC; Reverse, GAGGTGGCGTTTCTGGAATATAA.

### Western blot

Cells were lysed to obtain total protein by RIPA reagent (Jiancheng, Nanjing, China). After quantifying the protein concentration using a BCA protein assay kit (Thermo Fisher, CA, USA), the protein was separated by 10% SDS–PAGE and then transferred to polyvinylidene fluoride membranes (Millipore, MA, USA). Following blocking with 5% skim milk for 1 h, the membranes were incubated with primary antibodies (anti-Runx2, Abcam, ab192256; anti-Bglap, Proteintech, 20277-1-AP; anti-COL1A1, Proteintech, 67288-1-Ig; anti-bax, Proteintech, 60267-1-Ig; anti-bcl-2, Proteintech, 60178-1-Ig; anti-caspase3, Abcam, ab32042) overnight at 4°C. Next, the membranes were washed with TBST and incubated with horseradish peroxidase (HRP)-labeled secondary antibody (Abcam, ab6721). Protein bands were visualized using a gel imaging system (Bio-Rad, Hercules, USA).

### Pull-down assay with biotinylated miRNA

The interaction between miR-455-3p and circZfp644-205 or SMAD2 was assessed using RNA pull-down. Briefly, biotinylated probes were obtained from Sangon Biotech (CA, USA). Cells were transfected with circZfp644-205 probe, miR-455-3p probe, or their respective control probes. After 48 h, the cells were lysed and incubated with Dynabeads M-280 Streptavidin (Thermo Fisher Scientific) for 2 h according to the instructions of the supplier. The enrichment of miR-445-3p or SMAD2 in the pulled-down product was detected by qRT-PCR.

### Dual-luciferase reporter gene assay

The targeting relationship between miR-455-3p and circZfp644-205 or SMAD2 was confirmed using this experiment. Wild-type sequences of circZfp644-205 and SMAD2 containing potential miR-455-3p binding sites, as well as their mutant sequence, were synthesized by GENEWIZ (Suzhou, China) and then cloned into the psiCHECK2 reporter vectors (Promega, USA). MC3T3-E1 cells (4 × 10^4^ cells/well) were cultured overnight in 24-well plates. Subsequently, the cells were co-transfected with the wild-type or mutant psiCHECK2 reporter plasmids, along with miR-455-3p mimic or miR-455-3p control using Lipofectamine 2000 (Invitrogen, USA). After 24 h, firefly and Renilla luciferase activities were measured using a reporter gene assay system (Promega, USA), with Renilla luciferase activity serving as the endogenous control.

### Fluorescence in situ hybridization (FISH)

FISH was performed to observe the subcellular location of circZfnp644-205 using an in situ Hybridization kit (SIGMA, USA) according to the provided instructions. Briefly, cells were seeded onto 6-well plates. After fixation and permeabilization using 4% paraformaldehyde and 0.5% Triton X-100, respectively, the slides were incubated with the probe targeting circZfp664-205. DAPI was used to stain the cell nucleus. Images were taken under a fluorescence microscope (Nikon, Japan).

### Hindlimb unloading (HU) mouse model

All animal experiments conducted in this study were approved by the Animal Ethics and Experimental Safety Committee of Shanxi Provincial People’s Hospital and were performed according to the approved guidelines. The osteoporosis animal model was established as follows: male C57BL/6J mice (22.43 ± 0.97 g, Huafukang, Beijing, China) were suspended by the tail at an angle of approximately 30°, ensuring that their forelimbs could touch the floor to move and freely access food and water. Three weeks later, the mice were sacrificed, and bone specificity was evaluated using a Micro-CT scanner (Bruker, USA). A total of 40 mice were divided into 4 groups: control, HU model, HU+sh-nc, and HU+sh-circZfp644-205 groups. In the sh-nc and sh-circZfp644-205 treatment group, the mice were injected with 100 μL adenovirus (1*10^9^ TU/mL; Hanbio, Shanghai, China) expressing sh-nc or sh-circZfp644-205 via tail vein, respectively.

### Hematoxylin and eosin (H&E) staining assay

The distal femur samples acquired from the mice in each group were fixed with 4% paraformaldehyde for 48 h and decalcified using 10% EDTA for 30 days. Then, the samples were dehydrated with ethanol, permeabilized by xylene, embedded in paraffin, and cut into sections with 4 μm of thickness. After deparaffinizing and rehydrating, the sections were stained with hematoxylin, followed by eosin staining. The results are visualized under a microscope.

### Immunohistochemistry

The paraffin sections after deparaffinizing and rehydrating were treated with 3% H_2_O_2_ for 10 min to remove endogenous peroxidase. Next, antigen retrieval was performed by boiling using a citrate buffer solution. After blocking, the sections were incubated with anti-ALP (Abcam, ab224335) or anti-RUNX2 (Abcam, ab192256) overnight at 4 ℃, followed by incubation with the secondary antibody (Abcam, ab6721) at 37 ℃ for 0.5 h.

### Statistical analysis

Statistical analyses were performed using the SPSS software (version 17.0; SPSS Inc., Chicago, USA). All data were expressed as mean ± SD. Comparison between the two groups was determined by student’s *t* test. One-way ANOVA followed by Tukey’s post hoc test was used for comparison among different groups. *P* value less than 0.05 was considered to be significantly different.

## Results

### CircZfp644-205 expression was up-regulated in MG-treated MC3T3-E1 cells

**Fig. 1:**
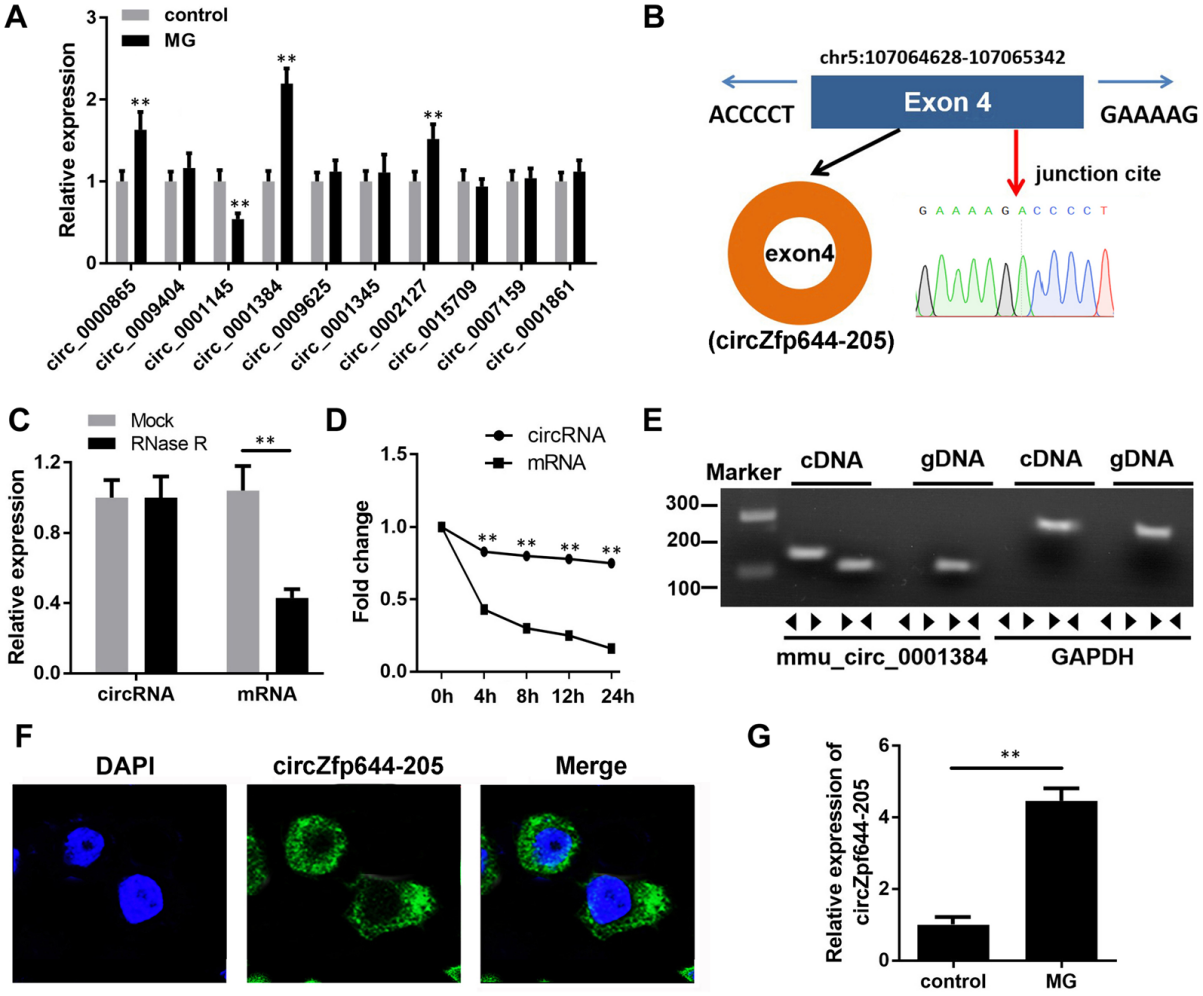
CircZfp644-205 expression was up-regulated in pre-osteoblasts after MG stimulation. **A** qRT-PCR was used to detect the expression of circRNAs in MC3T3-E1 cells after MG stimulation. **B** CircZfp644-205 was back-spliced by exons 4 of the *Zfp644* gene (black arrow). The existence of circZfp644-205 was confirmed by Sanger sequencing, and the red arrow shows the head-to-tail splicing junction site of circZfp644-205. **C** After RNase R treatment, the expression of linear mRNA Zfp644 and circZfp644-205 was detected by qRT-PCR. **D** qRT-PCR was employed to detect the mRNA expression of circZfp644-205 and linear Zfp644 in pre-osteoblasts after Actinomycin D treatment. **E** Existence of circZfp644-205 was validated by RT-PCR. Divergent primers amplified circZfp644-205 in cDNA but not in genomic DNA (gDNA). **F** FISH with a specific probe was performed to detect the subcellular localization of circZfp644-205. **G** qRT-PCR was used to evaluate the expression of circZpf644 in MC3T3-E1 cells treated with MG. **P < 0.01

To investigate which circular RNA participates in osteoporosis progression, we screened the dysregulated circRNAs in MG-treated MC3T3-E1 cells. The results indicated that circ_0001384 showed the most significant up-regulation (Fig. 1A**)**. This circRNA was looped by circularization of exon 4 of the *Zfp644* gene, located on chromosome 5 referring to the sequence from the circBase database (Fig. 1B). To avoid confusion with a previous study [15], this circRNA was named circZfp644-205. The head-to-tail splicing was further verified through Sanger sequencing. To assess the stability of circZfp644-205, its quantity was detected in the MC3T3-E1 cells after RNase R treatment. As shown in Fig. 1C, the amount of linear mRNA of Zfp644 was decreased significantly after RNase R treatment, while the expression of circZfp644-205 remained unchanged. Then, the half-lives of linear and circular *Zfp644* were further determined after treatment with actinomycin D. Results showed that the half-life of circZfp644-205 was longer than that of linear mRNA (Fig. 1D). In addition, cDNA and gDNA were extracted and subjected to nucleic acid electrophoresis detection. Results indicated that circZfp644-205 could be detected in cDNA but not in gDNA (Fig. 1E). Moreover, RNA FISH revealed that circZfp644-205 was mainly localized in the cytoplasm (Fig. 1F). These findings indicated the existence of circZfp644-205 in pre-osteoblasts. To investigate the role of circZfp644-205 during bone loss, we evaluated the expression of circZfp644-205 in the MC3T3-E1 cell under clinorotation conditions. We found that circZfp644-205 was notably up-regulated in the MC3T3-E1 cell under MG stimulation (Fig. 1G).

### CircZfp644-205 inhibits osteoblast differentiation and promotes apoptosis of MC3T3-E1 cells

**Fig. 2:**
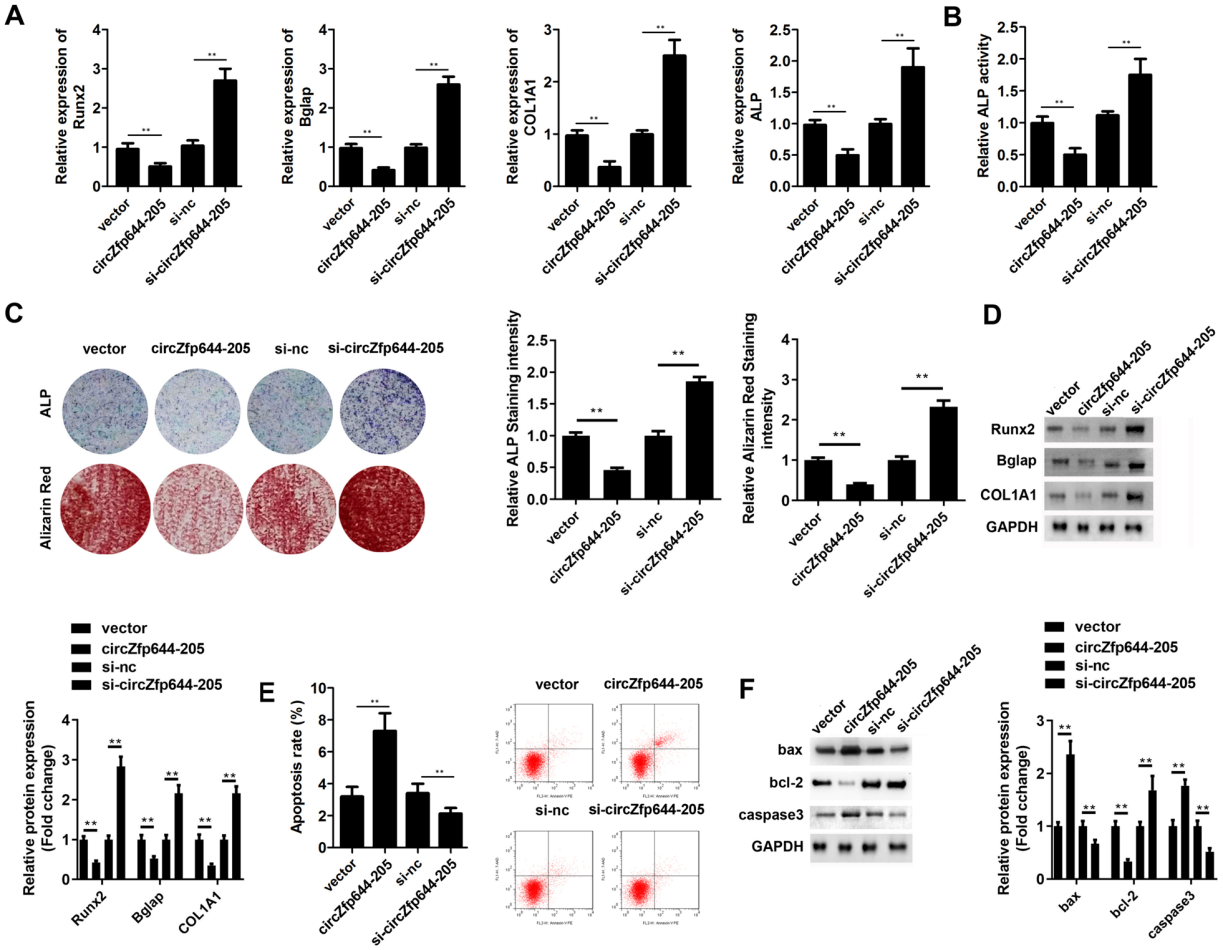
CircZfp644-205 inhibits osteogenic differentiation and promotes apoptosis of MC3T3-E1 cells. MC3T3-E1 cells were transfected with circZfp644-205 overexpressing vector or siRNA and then (**A**) qRT-PCR was used to detect the mRNA expression of osteogenic differentiation-related genes including Runx2, Bglap, COL1A1, and ALP. **B** ALP activity was measured by a kit. **C** ALP and ARS staining of MC3T3-E1 cells was performed to evaluate the osteogenic differentiation ability. Their relative staining intensities were quantified. **D** Western blot was used to detect the levels of osteogenic differentiation-related proteins, which were quantified. **E** Flow cytometry and Annexin V/PI staining were performed to detect the apoptosis of pre-osteoblasts. **F** Apoptosis markers (bax, caspase3, and bcl-2) were detected with western blot, and their levels were quantified. **P < 0.01

As circZfp644-205 expression was significantly up-regulated in MG-treated MC3T3-E1 cells, we speculated that circZfp644-205 might exert a critical role in osteogenic differentiation in osteoporosis. Thus, gain and loss of function studies were carried out. First, the mRNA expression of osteogenic biomarkers, including Runx2, Bglap, Col1a1, and ALP, in the pre-osteoblasts was detected. The results indicated that circZfp644-205 overexpression significantly inhibited the expression of these markers, while knockdown of circZfp644-205 increased their expression (Fig. 2A). Then, ALP activity was evaluated, and results showed that circZfp644-205 reduced the ALP activity, while knockdown of circZfp644-205 promoted the activity (Fig. 2B). ALP and ARS staining results indicated that circZfp644-205 inhibited the osteoblast differentiation activity of MC3T3-E1 cells (Fig. 2C). Western blot further supports these findings, revealing that circZfp644-205 significantly inhibited the protein expression of Runx2, Bglap, Col1a1, and ALP, whereas knockdown of circZfp644-205 promoted their levels (Fig. 2D). Flow cytometry was conducted to determine the apoptosis of MC3T3-E1 cells. Cell apoptosis was promoted by circZfp644-205 overexpression and was inhibited by circZfp644-205 knockdown (Fig. 2E). In addition, circZfp644-205 promoted the expression of Bax and caspase3, and inhibited that of bcl-2 (Fig. 2F).

### CircZfp644-205 sponged miR-455-3p in MC3T3-E1 cells

**Fig. 3:**
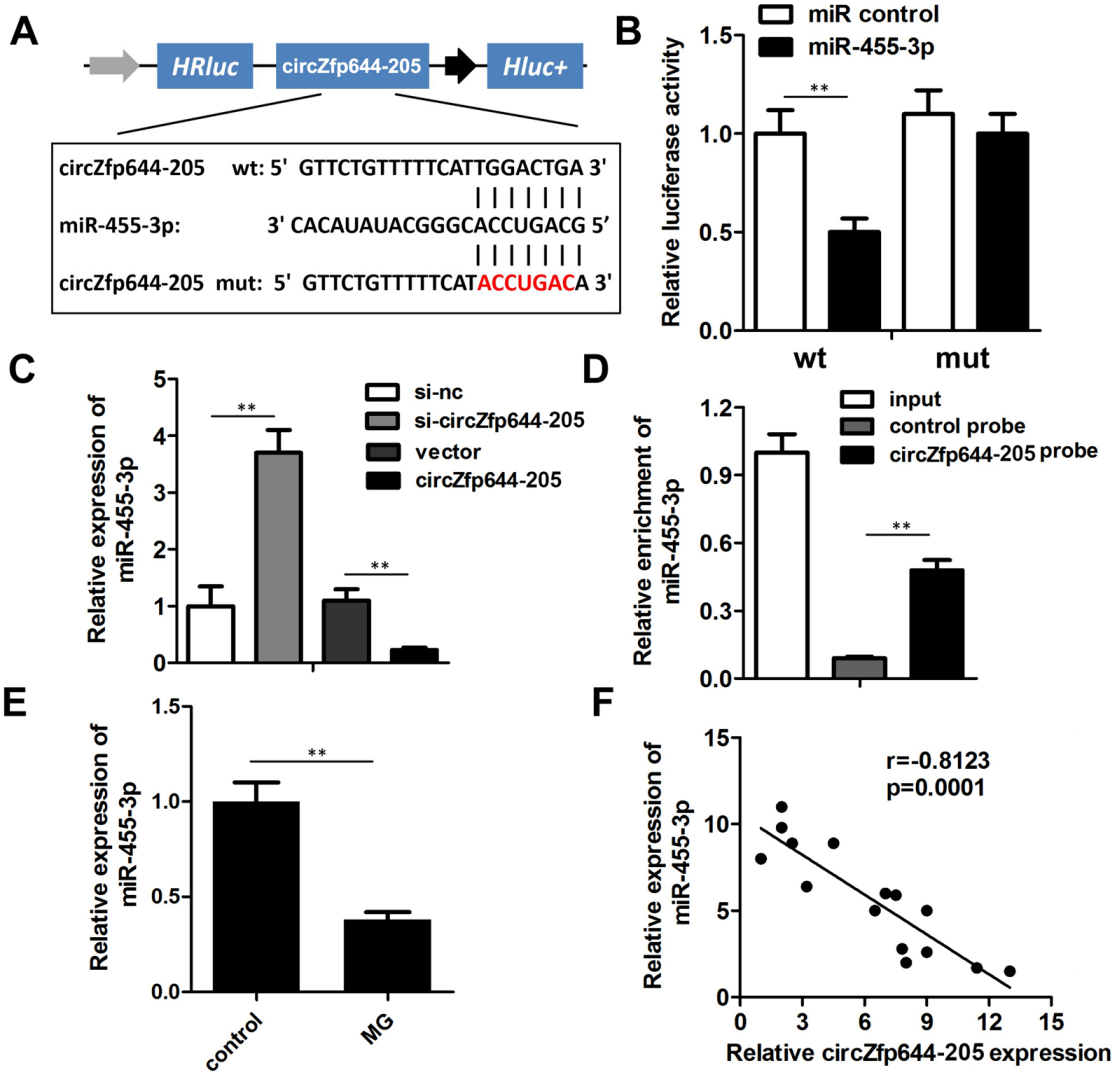
CircZfp644-205 sponged miR-455-3p in the pre-osteoblasts. **A** Wild type (wt) and mutant (mut) sequences of circZfp644-205 with the predicted targeting region of miR-455-3p were shown. **B** Luciferase assay was carried out to verify whether miR-455-3p targets circZfp644-205 in the pre-osteoblasts. **C** qRT-PCR was employed to detect the expression of miR-455-3p after circZfp644-205 overexpression or knockdown. **D** RNA pull-down with the specific probe of circZfp644-205 was used to confirm the interaction between circZfp644-205 and miR-455-3p. **E** qRT-PCR was used to evaluate the expression of miR-455-3p in MG-treated MC3T3-E1 cells. **F** Pearson analysis was used to detect the correlation between circZfp644-205 and miR-455-3p. **P < 0.01

It is well-known that circRNAs are capable of sponging miRNAs to subsequently inhibit the function of corresponding miRNAs. Thus, we investigated whether circZfp644-205 sponged miRNAs in pre-osteoblasts. miR-455-3p was predicted to be a potential miRNA target of circZfp644-205. Figure 3A shows the target region between circZfp644-205 and miR-455-3p. We conducted a luciferase assay in the pre-osteoblasts. As shown in Fig. 3B, miR-455-3p significantly down-regulated the luciferase activity after co-transfection with psiCHECK2-wt-circZfp644-205, but not with psiCHECK2-mut-circZfp644-205. qRT-PCR further confirmed that circZfp644-205 overexpression decreased the level of miR-455-3p, while knockdown of circZfp644-205 increased that of miR-455-3p (Fig. 3C). Moreover, RNA pull-down assay was carried out to assess whether circZfp644-205 could directly bind to miR-455-3p endogenously. Results showed that the circZfp644-205 probe enriched the expression of miR-455-3p (Fig. 3D). In addition, we detected the expression of miR-455-3p in the MC3T3-E1 cell under MG stimulation. We found that miR-455-3p was notably down-regulated in MG-induced MC3T3-E1 cells (Fig. 3E). Pearson analysis indicated a negative correlation between the expression of miR-455-3p and circZfp644-205 (Fig. 3F).

### miR-455-3p reversed the effect of circZfp644-205 on osteogenic differentiation and apoptosis of MC3T3-E1 cell

**Fig. 4:**
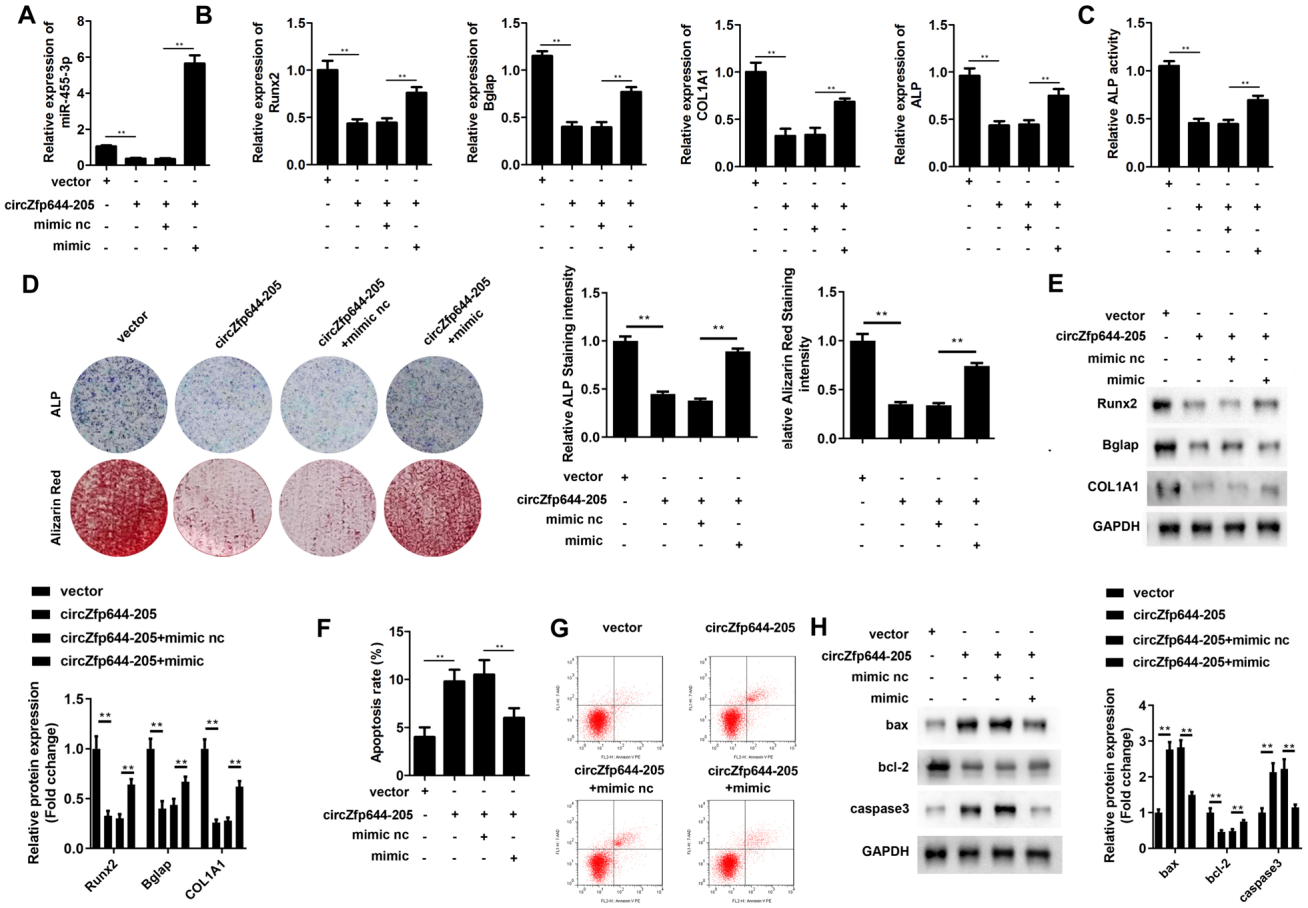
miR-455-3p reversed the effects of circZfp644-205 on osteogenic differentiation and apoptosis of pre-osteoblasts. After transfection, **A** qRT-PCR was used to evaluate the expression level of miR-455-3p in the pre-osteoblasts. **B** qRT-PCR was used to detect the mRNA expression of osteogenic differentiation-related genes including Runx2, Bglap, COL1A1, and ALP. **C** ALP activity was detected using a kit. **D** ALP and ARS staining of MC3T3-E1 cells. Relative staining intensities were quantified. **E** Western blot was used to detect the levels of osteogenic differentiation-related proteins, and their levels were quantified. **F**, **G** Flow cytometry and Annexin V/PI staining were performed to detect the apoptosis of pre-osteoblasts. **F** Apoptosis markers (bax, caspase3, and bcl-2) were detected with western blot, and their levels were quantified. **P < 0.01

Rescue experiments were performed to investigate the effect of miR-455-3p on osteogenic differentiation. MC3T3-E1 cells were transfected with circZfp644-205 overexpressing vectors and miR-455-3p mimics along with their negative controls, correspondingly. Co-transfection of circZfp644-205 and miR-455-3p resulted in an increase in the expression of miR-455-3p, compared to the circZfp644-205 transfection group (Fig. 4A). Subsequently, it was found that circZfp644-205 inhibited the mRNA expression of Runx2, Bglap, Col1a1, and ALP, while miR-455-3p reversed the effect on the mRNA expression mediated by circZfp644-205 (Fig. 4B). ALP activity results showed that circZfp644-205 inhibited the ALP activity of MC3T3-E1 cells, while miR-455-3p reversed this effect (Fig. 4C). ALP and ARS staining assays further revealed that miR-455-3p reversed the effect of circZfp644-205 on the osteogenic differentiation of MC3T3-E1 cells (Fig. 4D)**.** Western blot results showed that circZfp644-205 inhibited the expression of Runx2, Bglap, Col1a1, and ALP, while miR-455-3p reversed this inhibitory effect (Fig. 4E). The flow cytometry assay demonstrated that circZfp644-205 significantly increased the apoptosis rate of MC3T3-E1 cells, but miR-455-3p remarkably reversed this elevation (Fig. 4F, G). In addition, the evaluation of apoptosis-related proteins revealed that circZfp644-205 promoted the expression of Bax and caspase3 but inhibited that of bcl-2; however, miR-455-3p reversed their levels regulated by circZfp644-205 (Fig. 4H).

### miR-455-3p directly targeted SMAD2 in MC3T3-E1 cells

**Fig. 5:**
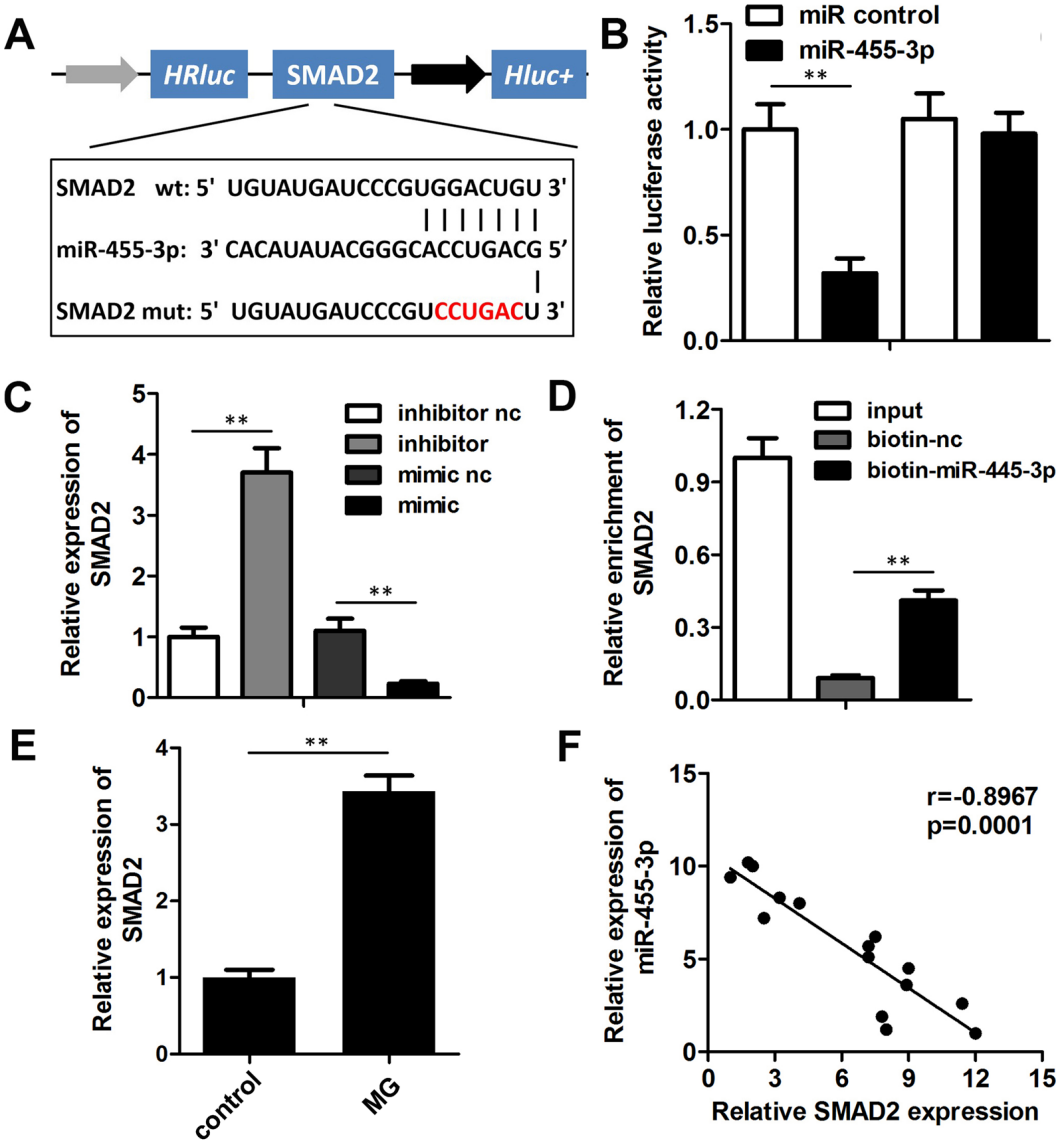
miR-455-3p directly targets SMAD2 in the pre-osteoblasts. **A** Bioinformatic analysis with the DIANA-TarBase database showed the target region between miR-455-3p and SMAD2. **B** Luciferase assay was carried out to verify whether miR-455-3p targets SMAD2 in the pre-osteoblasts. **C** qRT-PCR was performed to detect the mRNA expression of SMAD2 after miR-455-3p overexpression or knockdown. **D** RNA pull-down with the specific probe of miR-455-3p was used to confirm the interaction between miR-455-3p and SMAD2. **E** qRT-PCR was performed to detect the mRNA expression of SMAD2 in MG-treated MC3T3-E1 cells. **F** Pearson analysis was used to detect the correlation between miR-455-3p and SMAD2. **P < 0.01

The targets of miR-455-3p were predicted using the DIANA-TarBase database. Figure 5A shows the target region between miR-455-3p and SMAD2. A luciferase assay was performed in the pre-osteoblasts, and the results showed that miR-455-3p significantly down-regulated the luciferase activity after co-transfection with psiCHECK2-3’UTR of SMAD2, but not with psiCHECK2-3ʹUTR-mut (Fig. 5B). qRT-PCR further confirmed that miR-455-3p overexpression decreased the mRNA and protein expression of SMAD2, while knockdown of miR-455-3p up-regulated SMAD2 expression (Fig. 5C). RNA pull-down assay indicated that the miR-455-3p probe enriched the expression of SMAD2, suggesting a direct interaction between them (Fig. 5D). Moreover, the expression of SMAD2 was also detected in the MC3T3-E1 cell under MG stimulation, revealing a significant upregulation of SMAD2 expression (Fig. 5E). Finally, Pearson analysis was employed to assess the correlation between miR-455-3p and SMAD2, demonstrating a negative correlation between miR-455-3p and SMAD2 expression (Fig. 5F).

### Knockdown of SMAD2 reversed the effect of miR-455-3p on osteogenic differentiation and apoptosis of MC3T3-E1 cells

**Fig. 6:**
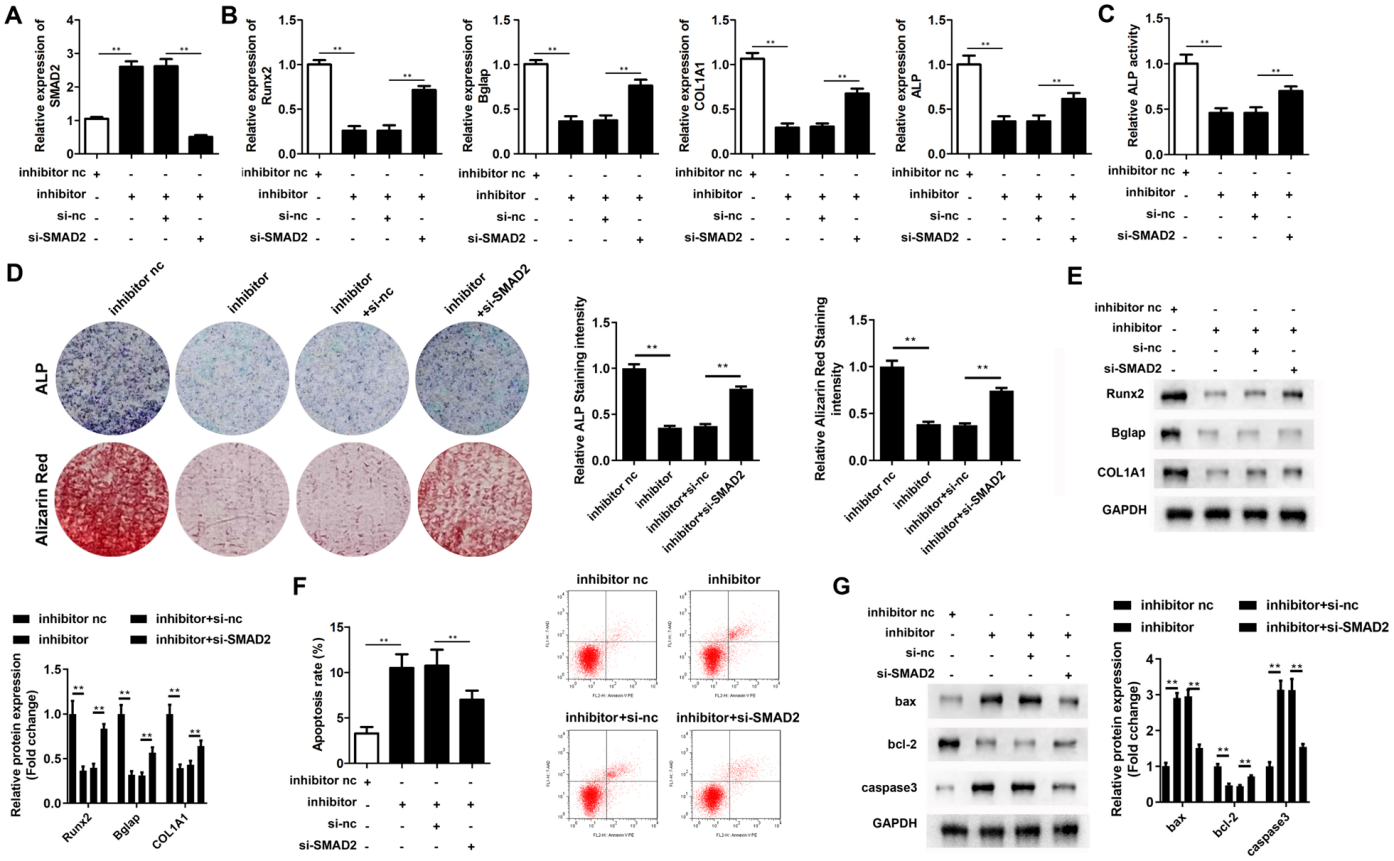
SMAD2 silencing reversed the effects of miR-455-3p knockdown on osteogenic differentiation and apoptosis of pre-osteoblasts. **A**, **B** qRT-PCR was used to evaluate the expression level of SMAD2 in the pre-osteoblasts. **B** qRT-PCR was used to detect the mRNA expression of osteogenic differentiation-related genes including Runx2, Bglap, COL1A1, and ALP. **C** ELISA was used to detect the ALP activity. **D** ALP and alizarin red staining of MC3T3-E1 cells, and their staining intensities were quantified. **E** Western blot was used to detect the levels of osteogenic differentiation-related proteins, and their levels were quantified. **F** Flow cytometry and Annexin V/PI staining were performed to detect the apoptosis of pre-osteoblasts. **G** Apoptosis markers (bax, cleaved caspase3, and bcl-2) were detected with western blot, and their levels were quantified. **P < 0.01

Rescue experiments were performed to investigate the role of miR-455-3p and SMAD2 in osteogenic differentiation. MC3T3-E1 cells were transfected with miR-455-3p inhibitor and si-SMAD2. Inhibition of miR-455-3p enhanced the expression level of SMAD2, while si-SMAD2 significantly reduced the level of SMAD2 (Fig. 6A). Downregulation of miR-455-3p reduced the expression levels of Runx2, Bglap, Col1a1, and ALP, as well as ALP activity, whereas SMAD2 knockdown reversed these effects (Fig. 6B, C, E). Moreover, miR-455-3p silencing inhibited the osteogenic differentiation activity of MC3T3-E1 cells, while SMAD2 knockdown reversed this effect (Fig. 6D). The flow cytometry results showed that silencing of miR-455-3p significantly increased the apoptosis rate of MC3T3-E1 cells, but SMAD2 knockdown remarkably reversed this elevation (Fig. 6F). In addition, the levels of apoptotic proteins including Bax and caspase3 were increased and bcl-2 was reduced after miR-455-3p downregulation, which was reversed by SMAD2 knockdown (Fig. 6G).

### CircZfp644-205 knockdown decelerated the progression of osteoporosis induced by HU

**Fig. 7:**
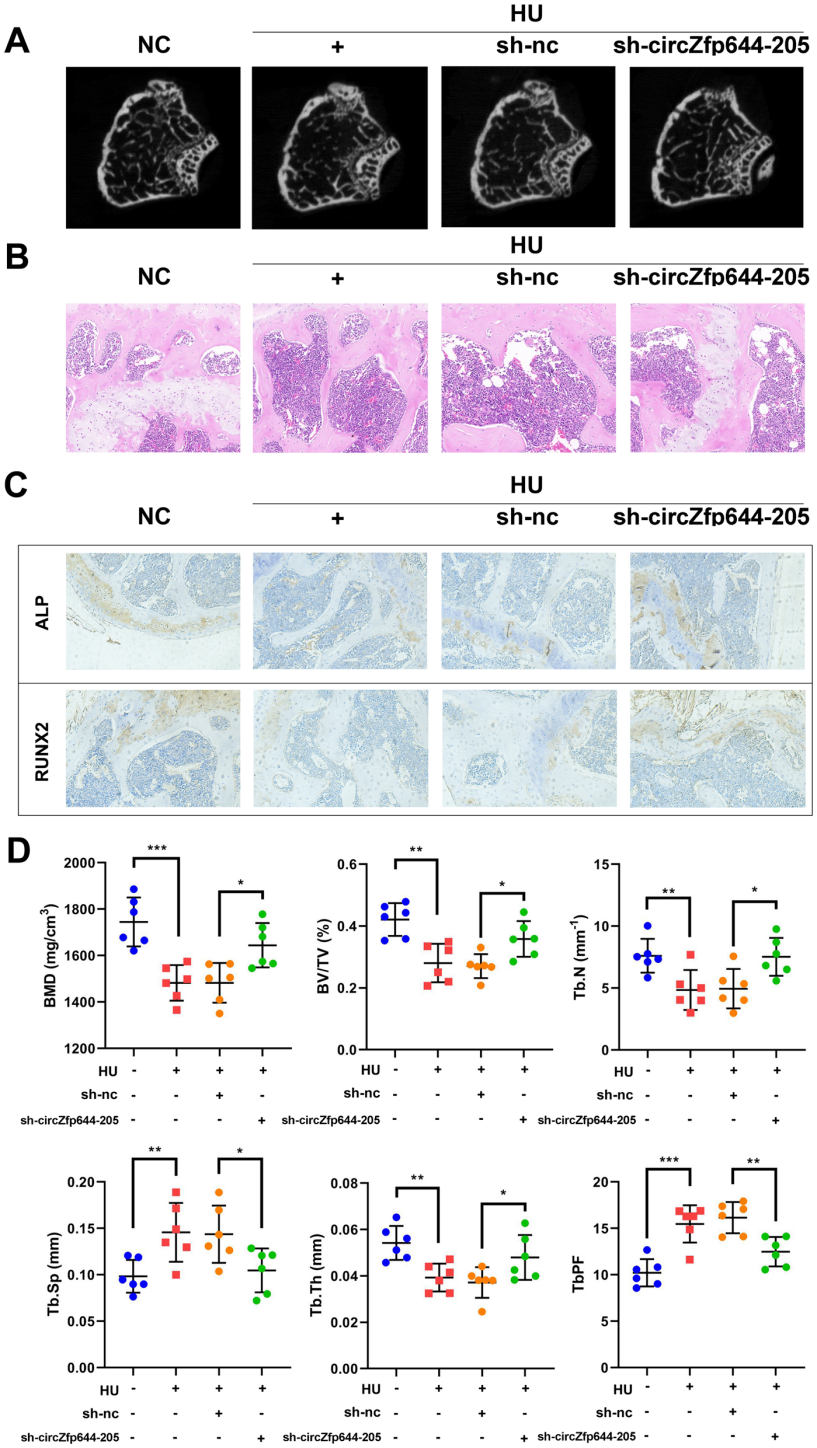
CircZfp644-205 knockdown improved bone phenotype in hindlimb unloading (HU) mice. **A** Micro-CT images of distal femurs of mice. **B** H&E staining images of distal femurs of mice. **C** ALP and RUNX2 levels in the femurs of mice were measured using immunohistochemistry. **D** BMD, BV/TV, Tb.N, Tb.Sp, Tb.Th, and Tb.PF were analyzed by Micro-CT. *P < 0.05, **P < 0.01, ***P < 0.001

After analyzing the regulation of circZfp644-205 in osteogenic differentiation and apoptosis, we next performed an animal study to further verify its role in osteoporosis. The Micro-CT analysis revealed that HU treatment significantly reduced the bone mineral density (BMD), and circZfp644-205 silencing increased it compared with the unloading model group. In addition, the ratio of bone volume to total volume (BV/TV), trabecular bone number (Tb.N), and trabecular thickness (Tb.Th) were reduced, while the bone trabecula separation (Tb. Sp) and trabecular bone pattern factor (Tb.PF) were increased after HU treatment. As expected, circZfp644-205 knockdown notably reversed these effects induced by HU (Fig. 7A, D). H&E staining results showed that HU induced bone loss in the femurs, whereas circZfp644-205 knockdown reversed these effects caused by HU (Fig. 7B). In addition, ALP and RUNX2 levels were downregulated in the femur samples from HU mice, which was reversed by silencing of circZfp644-205 (Fig. 7C). These findings confirmed that circZfp644-205 could decelerate the progression of osteoporosis.

## Discussion

Emerging evidence has shown that circRNAs play important roles in many biological processes, such as cell growth, proliferation, and invasion. However, the effects of circRNAs on osteogenic differentiation in osteoporosis have yet to be fully elucidated. Due to the stability of circular RNAs, they have the advantage of being molecular biomarkers. A previous clinical study has indicated that hsa_circ_0001275 serves as a potential diagnostic biomarker in postmenopausal osteoporosis [16]. Additional research has revealed that circRNAs are involved in the progression of osteoporosis. For example, circRNA_0048211 is known to protect against postmenopausal osteoporosis by targeting miRNA-93-5p to regulate BMP2. Hsa_circ_0076906 competes with OGN for the miR-1305 biding sites, thereby alleviating the progression of osteoporosis [17]. In the present study, we screened dysregulated circRNAs in MC3T3-E1 cells treated with MG, which is a cause of OP. The mechanism of this cell model is complex and controversial. Most studies indicate that MG may inhibit osteoblast activity by inducing apoptosis, thereby leading to bone loss [18–20]. Paradoxically, an additional study has shown that MG cannot directly induce osteoblast apoptosis [21]. However, despite the controversy surrounding this model affecting osteoblast activity, it is widely used in vitro to study the pathogenesis of osteoporosis [22–24]. A novel circRNA transcribed from exon 4 of the Zfp644 transcript was identified to be up-regulated in MG-treated cells, suggesting circZfp644-205 may play an important role in osteogenic differentiation. Further investigations revealed that circZfp644-205 could inhibit osteogenic differentiation and promote the apoptosis of pre-osteoblasts, and knockdown of circZfp644-205 alleviated osteoporosis in vivo. The findings suggest that circZfp644-205 aggravates osteoporosis by inhibiting osteogenic differentiation and inducing apoptosis.

It is well-known that circRNAs can act as sponges of miRNAs, participating in the regulation of their target genes that are involved in common cellular processes [25–27]. Studies have reported that circ_0076906 [17], circ_0048211 [28], circ_28313 [29], and circ_0016624 [5] mediate I/R injury through sponging miR-1305, 93a-5p, miR-195a and miR-98, respectively. To understand the critical role of circZfp644-205 in osteogenic differentiation, further investigation was conducted into the molecular mechanisms by which circZfp644-205 exerts its effects. Bioinformatic analysis predicted that circZfp644-205 contains binding sites in miR-455-3p, and luciferase activity assay confirmed circZfp644-205 as a sponge of miR-455-3p.

Increasing evidence has demonstrated that miR-455-3p plays regulatory functions in many human diseases. It functions as a tumor promoter or inhibitor in various types of tumors, including breast, pancreas, esophagus, colon, and lung cancers [30–33]. In addition, it also plays a critical role in the progression of renal fibrosis [34], pulmonary arterial hypertension [35], cartilage degeneration [36], ischemic brain injury [37], and heart failure. Interestingly, miR-455-3p has been confirmed to promote pre-osteoblast growth via modulating the Nrf2/ARE signal pathway [38]. In this study, we found that miR-455-3p expression was down-regulated in MC3T3-E1 cells under MG conditions. MiR-455-3p reversed the inhibition of circZfp644-205 on the osteogenic differentiation, suggesting that miR-455-3p promotes osteogenic differentiation, consistent with the previous study [39].

Further investigations indicated that miR-455-3p directly targets SMAD2 in pre-osteoblasts, and their interaction was confirmed. SMAD proteins, including SMAD 1, 2, 3, 5, and 8, act as critical intracellular receptors and play a role in canonical TGF-β and BMP superfamily pathways [40]. Among the SMAD proteins, SMAD2/3/4 are the major transcription factors of TGF-β1 signaling and are involved in TGF-β-related cell responses and pathophysiological functions. It is well-known that the TGF-β/Smad signaling plays a crucial role in modulating osteoblast differentiation [41]. For instance, fish bone peptide promotes osteogenic differentiation of MC3T3-E1 pre-osteoblasts through upregulation of the SMAD pathways activated BMP-2 receptor [42]. Our results first showed that SMAD2 was a target gene of miR-455-3p in the pre-osteoblasts, thereby participating in the ceRNA mechanism of circZfp644-205. SMAD2 directly regulated osteogenic differentiation and apoptosis. However, the expression of other proteins in the TGF-β/SMAD signal pathway has not been investigated. The precise mechanism remains to be elucidated through further research work.

## Conclusions

In the present study, we have identified a novel circRNA which was named circZfp644-205. The expression of circZfp644-205 is up-regulated following MG treatment. CircZfp644-205 inhibited osteogenic differentiation and induced the apoptosis of pre-osteoblasts. In addition, circZfp644-205 acts as a sponge of miR-455-3p, which directly targets SMAD2 to participate in the regulation of osteogenic differentiation. These findings provide a novel potential target for the treatment of osteoporosis.

## Data Availability

The data sets used and/or analysed during the current study are available from the corresponding author on reasonable request.

## References

[1] Liu T, Guo J (2020). Overexpression of microRNA-141 inhibits osteoporosis in the jawbones of ovariectomized rats by regulating the Wnt/β-catenin pathway. Arch Oral Biol.

[2] Koyama C, Hirota M, Okamoto Y, Iwai T, Ogawa T, Hayakawa T (2020). A nitrogen-containing bisphosphonate inhibits osteoblast attachment and impairs bone healing in bone-compatible scaffold. J Mech Behav Biomed.

[3] Zhang R, Wesevich V, Chen Z, Zhang D, Kallen AN (2020). Emerging roles for noncoding RNAs in female sex steroids and reproductive disease. Mol Cell Endocrinol.

[4] Suenkel C, Cavalli D, Massalini S, Calegari F, Rajewsky N (2020). A highly conserved circular RNA is required to keep neural cells in a progenitor state in the mammalian brain. Cell Rep.

[5] Yu L, Liu Y (2019). circRNA_0016624 could sponge miR-98 to regulate BMP2 expression in postmenopausal osteoporosis. Biochem Bioph Res Co.

[6] Wang XB, Li PB, Guo SF, Yang QS, Chen ZX, Wang D (2019). circRNA_0006393 promotes osteogenesis in glucocorticoidinduced osteoporosis by sponging miR1455p and upregulating FOXO1. Mol Med Rep.

[7] Cao G, Meng X, Han X, Li J (2020). Exosomes derived from circRNA Rtn4-modified BMSCs attenuate TNF-alpha-induced cytotoxicity and apoptosis in murine MC3T3-E1 cells by sponging miR-146a. Bioscience Rep.

[8] Huang X, Cen X, Zhang B, Liao Y, Zhu G, Liu J (2019). Prospect of circular RNA in osteogenesis: a novel orchestrator of signaling pathways. J Cell Physiol.

[9] Huang Y, Xie J, Li E (2019). Comprehensive circular RNA profiling reveals circ_0002060 as a potential diagnostic biomarkers for osteoporosis. J Cell Biochem.

[10] Herrmann M, Engelke K, Ebert R, Muller-Deubert S, Rudert M, Ziouti F (2020). Interactions between muscle and bone-where physics meets biology. Biomolecules.

[11] Bonanni R, Cariati I, Marini M, Tarantino U, Tancredi V (2023). Microgravity and musculoskeletal health: what strategies should be used for a great challenge?. Life.

[12] Cao Q, Zhang J, Liu H, Wu Q, Chen J, Chen GQ (2014). The mechanism of anti-osteoporosis effects of 3-hydroxybutyrate and derivatives under simulated microgravity. Biomaterials.

[13] Komori T (2015). Animal models for osteoporosis. Eur J Pharmacol.

[14] Cheng J, Zhuo H, Wang L, Zheng W, Chen X, Hou J (2020). Identification of the combinatorial effect of mirna family regulatory network in different growth patterns of GC. Mol Ther-Oncolytics.

[15] Yang Y, Ren J, Huang Q, Wu J, Yuan X, Jiang W (2020). CircRNA expression profiles and the potential role of CircZFP644 in mice with severe acute pancreatitis via sponging miR-21-3p. Front Genet.

[16] Zhao K, Zhao Q, Guo Z, Chen Z, Hu Y, Su J (2018). Hsa_Circ_0001275: a potential novel diagnostic biomarker for postmenopausal osteoporosis. Cell Physiol Biochem.

[17] Wen J, Guan Z, Yu B, Guo J, Shi Y, Hu L (2020). Circular RNA hsa_circ_0076906 competes with OGN for miR-1305 biding site to alleviate the progression of osteoporosis. Int J Biochem Cell B.

[18] Bucaro MA, Fertala J, Adams CS, Steinbeck M, Ayyaswamy P, Mukundakrishnan K (2004). Bone cell survival in microgravity: evidence that modeled microgravity increases osteoblast sensitivity to apoptogens. Ann NY Acad Sci.

[19] Mukundakrishnan K, Ayyaswamy PS, Risbud M, Hu HH, Shapiro IM (2004). Modeling of phosphate ion transfer to the surface of osteoblasts under normal gravity and simulated microgravity conditions. Ann NY Acad Sci.

[20] Yuge L, Hide I, Kumagai T, Kumei Y, Takeda S, Kanno M (2003). Cell differentiation and p38(MAPK) cascade are inhibited in human osteoblasts cultured in a three-dimensional clinostat. In Vitro Cell Dev-An.

[21] Bucaro MA, Zahm AM, Risbud MV, Ayyaswamy PS, Mukundakrishnan K, Steinbeck MJ (2007). The effect of simulated microgravity on osteoblasts is independent of the induction of apoptosis. J Cell Biochem.

[22] Yoo YM, Han TY, Kim HS (2016). Melatonin suppresses autophagy induced by clinostat in preosteoblast MC3T3-E1 Cells. Int J Mol Sci.

[23] Zhou Y, Xu Z, Wang Y, Song Q, Yin R (2022). LncRNA MALAT1 mediates osteogenic differentiation in osteoporosis by regulating the miR-485-5p/WNT7B axis. Front Endocrinol.

[24] Wang Y, Wang K, Hu Z, Zhou H, Zhang L, Wang H (2018). MicroRNA-139-3p regulates osteoblast differentiation and apoptosis by targeting ELK1 and interacting with long noncoding RNA ODSM. Cell Death Dis.

[25] Szabo L, Salzman J (2016). Detecting circular RNAs: bioinformatic and experimental challenges. Nat Rev Genet.

[26] Panda AC (2018). Circular RNAs Act as miRNA sponges. Adv Exp MED BIOL.

[27] Zhang Z, Yang T, Xiao J (2018). Circular RNAs: promising biomarkers for human diseases. EBioMedicine.

[28] Qiao L, Li CG, Liu D (2020). CircRNA_0048211 protects postmenopausal osteoporosis through targeting miRNA-93-5p to regulate BMP2. Eur Rev Med Pharmaco.

[29] Chen X, Ouyang Z, Shen Y, Liu B, Zhang Q, Wan L (2019). CircRNA_28313/miR-195a/CSF1 axis modulates osteoclast differentiation to affect OVX-induced bone absorption in mice. RNA Biol.

[30] Guo J, Liu C, Wang W, Liu Y, He H, Chen C (2018). Identification of serum miR-1915-3p and miR-455-3p as biomarkers for breast cancer. PLoS ONE.

[31] Liu A, Zhu J, Wu G, Cao L, Tan Z, Zhang S (2017). Antagonizing miR-455-3p inhibits chemoresistance and aggressiveness in esophageal squamous cell carcinoma. Mol Cancer.

[32] Zhan T, Huang X, Tian X, Chen X, Ding Y, Luo H (2018). Downregulation of MicroRNA-455-3p links to proliferation and drug resistance of pancreatic cancer cells via targeting TAZ. Mol Ther-Nucl Acids.

[33] Zheng J, Lin Z, Zhang L, Chen H (2016). MicroRNA-455-3p inhibits tumor cell proliferation and induces apoptosis in HCT116 human colon cancer cells. Med Sci Monitor.

[34] Wu J, Liu J, Ding Y, Zhu M, Lu K, Zhou J (2018). MiR-455-3p suppresses renal fibrosis through repression of ROCK2 expression in diabetic nephropathy. Biochem Bioph Res Co.

[35] Zhou C, Chen Y, Kang W, Lv H, Fang Z, Yan F (2019). Mir-455-3p-1 represses FGF7 expression to inhibit pulmonary arterial hypertension through inhibiting the RAS/ERK signaling pathway. J Mol Cell Cardiol.

[36] Hu S, Zhao X, Mao G, Zhang Z, Wen X, Zhang C (2019). MicroRNA-455-3p promotes TGF-beta signaling and inhibits osteoarthritis development by directly targeting PAK2. Exp Mol Med.

[37] Zhou Y, Chai X (2020). Protective effect of bicyclol against pulmonary fibrosis via regulation of microRNA-455-3p in rats. J Cell Biochem.

[38] Zhang S, Wu W, Jiao G, Li C, Liu H (2018). MiR-455-3p activates Nrf2/ARE signaling via HDAC2 and protects osteoblasts from oxidative stress. Int J Biol Macromol.

[39] Ma H, Li M, Jia Z, Chen X, Bu N (2022). MicroRNA-455-3p promotes osteoblast differentiation via targeting HDAC2. Injury.

[40] Song B, Estrada KD, Lyons KM (2009). Smad signaling in skeletal development and regeneration. Cytokine Growth F R.

[41] Witkowska M, Smolewski P (2014). SMAD family proteins: the current knowledge on their expression and potential role in neoplastic diseases. Postep Hig Med Dosw.

[42] Heo SY, Ko SC, Nam SY, Oh J, Kim YM, Kim JI (2018). Fish bone peptide promotes osteogenic differentiation of MC3T3-E1 pre-osteoblasts through upregulation of MAPKs and Smad pathways activated BMP-2 receptor. Cell Biochem Funct.

